# Some of the Dangers of an Impure Meat-Supply and Milk-Supply1Read before the Louisiana State Sanitary Association at Baton Rouge, June, 1899.

**Published:** 1900-06

**Authors:** W. H. Dalrymple

**Affiliations:** Baton Rouge, La.


### SOME OF THE DANGERS OF AN IMPURE MEAT-SUPPLY AND MILK-SUPPLY.[1]

By W. H. Dalrymple, M.R.C.V.S.,
BATON ROUGE, LA.

(Concluded from page 264.)

In order that I may not occupy too much time in discussing this subject, although it is one of intense moment, I will take the liberty of quoting a few statistics recently given in a valuable paper by an English medical officer of health, and which I trust may serve the purpose of emphasizing the fact that there is no lack of evidence of the causation of human tuberculosis by cow's milk. Among other things Dr. Harold Scurfield (the gentleman referred to), medical officer for Sunderland, a large town in the north of England, says: "There are two reasons why the eradication of bovine tuberculosis is desirable. First, in the interest of agricultural communities; and, second, because its eradication necessarily entails a large reduction in the prevalence of one of the most fatal diseases of childhood, namely, tuberculosis or consumption of the bowels."

[1] Read before the Louisiana State Sanitary Association at Baton Rouge, June, 1899.

In enumerating some of the evidence as to the causation of human tuberculosis by cow's milk, Dr. Scurfield cites the following few striking features in the chain: (1) Tuberculosis in cattle and man is the same disease, and is caused by the entrance into and growth in the body of the tubercle bacillus. The entrance of the bacillus into the body is so rarely effected before birth that for all practical purposes it is true to say that every case of tuberculosis is due to infection. And only a day or two ago, at the tuberculosis congress held at Berlin, Professor Rudolph Virchow, in an address before that body, rejected the theory of hereditary tuberculosis. This doctrine, he declared, was contradicted by all his pathological researches. He said he had never found tuberculosis in unborn or new-born infants, though it may be contracted during the first day's existence. (2) Cows suffering from tuberculosis of the udder give milk containing tubercle bacilli. (3) Tuberculosis of the udder is not rare in cows, and milk, as ordinarily sold, often contains tubercle bacilli. Thus Bang, of Copenhagen, says: "In any large herd in which tuberculosis is rife, and which has tuberculosis of the udder, one will be certain to find one cow the milk of which contains tubercle bacilli."

Out of 144 samples of milk coming from Liverpool cow stables, four, or 2.8 per cent., were found to contain the germ of consumption, but of twenty-four samples of "country milk" taken at Liverpool railroad depots, seven, or 29 per cent., were found to contain the organism.

In some later experiments in Liverpool, twelve out of 228 samples of city milk (5.2 per cent.) were found to contain the germ, and nine out of sixty-seven samples of country milk or 13.4 per cent.

Similar results were obtained at Cambridge. In Manchester seventeen out of ninety-three samples of country milk were found to contain the bacillus. By permission of the farmers the city veterinary officer visited sixteen farms from which the milk came, and on fourteen of these farms he found at least one cow with tuberculosis of the udder.

(4) Animals such as calves, hogs, rabbits, guinea-pigs, cats, and dogs fed on milk from cows suffering from tuberculosis of the udder develop tuberculosis of the bowels. (5) Human beings contract consumption of the bowels just at the period of life when cow's milk forms the principal item in their diet. (6) The sanitary improvements of the last forty years have reduced the death-rate from tuberculosis of the lungs by 45 per cent., or nearly one-half.

During the same period the death-rate from consumption of the bowels among infants under one year old has increased by 27 per cent., and this increase has been coincident with an increase in the proportion of infants raised on the bottle instead of on their natural nutriment. Tuberculosis of the breast is rare in the human mother, but tuberculosis of the udder is comparatively common in the cow.

We have, then, to contemplate the suggestive fact that the transfer of the infant from the rarely tuberculous breast of the mother to the frequently tubercular udder of the cow has been coincident with an increase in the prevalence of tuberculosis of the bowels, and that this increase has taken place during a period when the prevalence of other forms of tuberculosis in the human being have been rapidly decreasing.

Such facts as these, and we could find plenty more evidence to back them up here in our own country, should impress themselves not only upon the minds of those who have charge of the health of our citizens, but on the citizens themselves, for, as I have previously stated, it is only by concerted effort that the necessity for such important reforms as indicated by these statements can be sufficiently appreciated or their achievement made an established fact.

How, then, are we to bring about the much-needed reforms and secure for our citizens a pure and wholesome meat-supply and milk-supply? The matter has received a great amount of attention and study by the leading sanitarians both of this country and of Europe, with the result that in the most progressive centres of population of both continents it has been found necessary, in order to obtain the greatest amount of success, to consolidate slaughter-houses into one or more—according to requirements—central abattoirs placed under municipal control, the dairies also supplying city populations under similar control, with inspection of both placed in the hands of an individual or individuals, as the requirements may demand, who has been specially trained for such work.

What would be the effect of such a system? it might be asked. We have but to look at the records of other cities that have adopted it to find that it has proved itself a boon to the consumer and a benefit to the vender. Those who are engaged in providing meat and milk for human consumption, and especially in our centres of population, are, generally speaking, citizens of the highest honor and integrity, and would not, if they were aware of it, place on the market for human food either meat or milk that they did not consider perfectly sound and nutritious; but for lack of the necessary pathological knowledge the purveyor of these articles is unable to identify conditions which would be at once observed by the trained eye of the expert, and known by him to be injurious. Besides, the former has, as a rule, little or no appreciation of the great importance from a public health stand-point of rigid sanitary conditions and surroundings, and even if he did he has not the facilities for such in the ordinary butcher-pen or the common cow stable or dairy. Nor does he realize the danger of the contamination of his products resulting from insanitation, whereas the expert, who has been trained in hygiene and sanitation, is at once familiar with the various agencies and pathological conditions that are likely to bring about disease. How much better and more satisfactory it must be for the butcher, who is an honorable man and not afraid to expose his methods of work or his wares, to have his work done in an abattoir fitted up with all the most modern improvements and appliances to ensure speed and cleanliness than to labor in a place the whereabouts of which can often be easily discovered without the aid of a guide; or the milk vender who is cognizant of the fact by actual test that his cows are free from tuberculosis, and that his milk has been handled under the most approved sanitary precautions and reaches the customer positively pure and wholesome. And how much more comfortable must be the feelings of the consumer who sits down with his family to his roast of beef or pork or other meat products, or his pitcher of milk, when he knows that they have been produced by animals that are not only free from disease but in a fit condition to slaughter for food or to yield milk for consumption, and that they have been handled with the greatest sanitary care; have passed before the scrutinizing eye of a competent inspector, and come to his table stamped with the mark of purity on them.

I have not the time on this occasion to go into the details with regard to the construction and working of a public abattoir or a meat-inspection and milk-inspection service under municipal control. My object at present is chiefly to set before you a few of the too-frequently overlooked dangers to public health, which frequently lurk in the flesh of some of the more important of our meat animals, and in the milk of our dairy herds, that you may the more fully appreciate the necessity for the inauguration of a system where it does not already exist that will ensure to the consumer these valuable food materials in an absolutely pure and wholesome condition.

It is only a matter of time when every city and town of any size in the country will have its public abattoir with its meat-inspection and milk-inspection under municipal control. Public opinion is fast being educated along this particular line; and when it becomes more generally known that some important diseases can be transmitted to human beings through the consumption of unsound meat and milk under the old and in many cases the present unsanitary system, and that such diseases can be positively prevented by adopting the new and more sanitary system, the latter is going to be demanded.

Outside of the city of New Orleans, which has had a meat-inspection and milk-inspection for several years, the city of Shreveport is taking the lead. She is now engaged, or is about to commence, in the erection of a modern building to be under the control of the City Board of Health, where all animals that are intended to supply meat to their citizens will have to be slaughtered and their flesh inspected before being allowed to be placed on the market as an article of human food. This will ensure to them a meat-supply that is not only free from disease but also a food that is nutritious and wholesome.

Why, I might ask, should our capital city be behind the city of Shreveport? Our citizens are quite as progressive, I hope, and I feel sure they are just as epicurean in their tastes. This subject should in reality enlist the serious consideration of the citizens of every community in the State; but it appears to me to be extremely opportune that we should devote to it special thought at the present time. We have in contemplation extensive improvements for our city along sanitary lines. We have just had the indorsement of our citizens to the imposition of a tax for the purpose of securing sufficient money to carry out these improvements.

Why not add to the list a public abattoir and the other necessaries required to ensure a meat-supply and milk-supply that would not only be pure and wholesome, but for quality, also, would be second to none in the country? It would be a lasting monument to the intelligence and progressiveness of the citizens of our town, and with the result that no one would suffer financially but all would benefit from the all-important stand-point of public health.

---

The successful use of carbolic acid in tetanus in the human family is attracting much attention in medical circles. Its favorable action in veterinary practice has attracted much attention for several years, and many veterinarians have reported successful results in a number of cases.

